# Clinical, haematological and pathomorphological findings in *Mycoplasma suis* infected pigs

**DOI:** 10.1186/s12917-021-02919-5

**Published:** 2021-06-10

**Authors:** Julia Stadler, Julia Ade, Walter Hermanns, Mathias Ritzmann, Sarah Wentzel, Katharina Hoelzle, Ludwig E. Hoelzle

**Affiliations:** 1 Clinic for Swine, Centre for Clinical Veterinary Medicine, Ludwig-Maximilians-University Munich, Sonnenstr. 16, 85764 Oberschleissheim, Germany; 2 Institute of Veterinary Pathology, Centre for Clinical Veterinary Medicine, Ludwig-Maximilians-University Munich, Munich, Germany; 3 Institute of Animal Science, University of Hohenheim, Garbenstrasse 30, 70593 Stuttgart, Germany

**Keywords:** Haemotrophic mycoplasmas, *Mycoplasma suis*, Histopathological findings, Tissue sequestration

## Abstract

**Abstract:**

**Background:**

*Mycoplasma suis* (*M. suis*) belongs to the group of haemotrophic mycoplasmas and is known as the causative agent of infectious anaemia in pigs. In the last few years valuable insights into the mechanism of adhesion and invasion, shedding patterns and cell tropism of *M. suis* were gained by the use of new molecular techniques. However, details on *M. suis* induced lesions as well as the distribution of *M. suis* in different organs are still lacking. Therefore, seven splenectomised pigs were experimentally infected and clinical and laboratory investigations as well as a detailed histopathological examination were performed. Detection and quantification of *M. suis* DNA in blood and various tissue samples was done using a quantitative real-time PCR.

**Results:**

During the course of experimental infection, periodically occurring signs of infectious anaemia of pigs including severe icteroanaemia, fever, apathy and anorexia were observed. In addition, dermatological manifestations such as haemorrhagic diathesis presenting as petechiae occurred. The most important haematological alterations were normochromic, normocytic anaemia, hypoglycaemia as well as increased bilirubin and urea concentrations. Necropsy revealed predominant evidence of haemolysis with consecutive anaemia, as well as disseminated intravascular coagulation. *M. suis* was found in all investigated tissues with the highest copy numbers found in the kidneys. In Giemsa stained sections *M. suis* was only detected red blood cell (RBC)-associated.

**Conclusion:**

In the present study, no RBC independent sequestration of *M. suis* was detected in organs of experimentally infected pigs. Pathological findings are most likely resulting from haemolysis, consecutive anaemia as well as from disseminated intravascular coagulation and subsequent organ impairments.

## Background

*Mycoplasma suis* is a so far uncultivable haemotrophic pathogen that causes acute or chronic infectious anaemia in pigs (IAP) worldwide [1–5]. Clinical manifestation of IAP depends on many factors such as the animal’s immune status, the age of the animal, the infection dose and the strain’s virulence [2, 3, 6]. Acute infections are mainly found in piglets and feeder pigs and manifest as life-threatening haemolytic anaemia, high fever, icterus and hypoglycaemia. Chronic infections can cause a variety of clinical signs including mild anaemia, skin alterations, performance depressions concerning fattening and reproduction parameters and immune suppression leading to an increased susceptibility to respiratory and intestinal infections [1, 7–11]. Up to date, transmission of *M. suis* via parenteral exposure during zootechnical procedures or through ranking fights have been identified as key epidemiological routes [2, 9, 11]. Pathological lesions induced by *M. suis* are rarely and incompletely described in literature.

*M. suis*is primary found in the blood on the surface of red blood cells (RBCs), inside RBCs or, to a lesser extent, free in the plasma [3, 6]. In addition, a tropism of *M. suis* to the endothelium has also been proven so far [3, 12].

Establishment of persistent infections as well as fluctuating bacteraemia are hallmarks of haemoplasma infections [1, 3, 13]. Experimental studies have shown that *M. suis* infected animals reached maximal copy numbers of 10^5^ to 10^10^ *M. suis*/mL blood within 6–7 days post infection (dpi) in splenectomised pigs [10] and maximum copy numbers of 10^3^ to 10^9^ copies/ mL blood within 23 to 30 dpi in intact animals [14]. After antibiotic treatment, a rapid and marked decline of *M. suis* occurs within 1 to 2 days [10, 15, 16] but treatment does not eliminate the pathogen. Experimentally infected, non-splenectomised pigs are known to develop transiently qPCR negative episodes for several days followed by recurrent *M. suis* bacteraemia [10, 14]. Similar infection courses were described in endemically infected pigs which develop acute forms of the disease with high bacterial blood loads after immunosuppressive events (i.e. prepartum, weaning or environmental stress) [1]. So far, little is known about the mechanisms behind the development of persistence and cyclic episodes of bacterial blood loads in *M. suis* infections. Possibly, propagation as well as sequestration of *M. suis* in organs and a subsequent pathogen release under immunosuppressive conditions may be involved in the largely unknown pathogenesis.

Thus, the aim of the present study was to investigate the possible RBC-independent sequestration of *M. suis* in different organs as well as tissue alterations due to *M. suis* in experimentally infected pigs in order to identify potential *M. suis* target tissues and sequestration sites which could be involved in IAP pathogenesis.

## Results

### Clinical observations

**Fig. 1:**
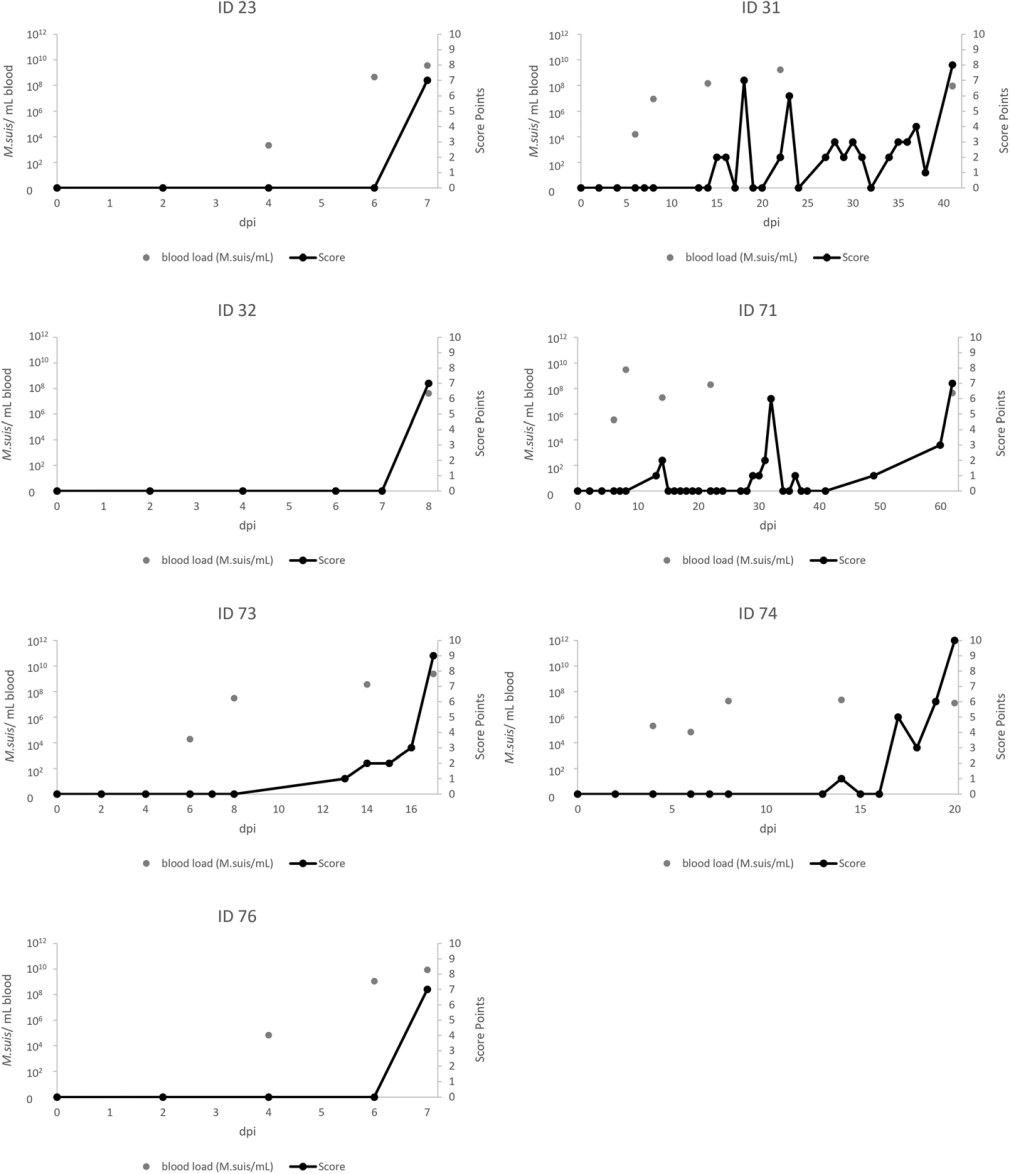
Clinical examination and *M. suis* blood loads. Score points of the daily clinical examination and *M. suis* blood loads (*M. suis*/mL) of all seven animals during the entire study period. *X*-axis indicates DPI (days post infection)

On 7 and 8 dpi, three animals (ID 23; 32; 76) showed clinical signs of acute IAP including impaired general condition, fever up to 41.4 °C, apathy, anorexia and skin alterations described for *M. suis* infections such as icterus and haemorrhagic diathesis presenting as petechiae and cyanosis of the ear tips. As those three animals (ID 23; 32, 76) fulfilled the abortion criteria of the scoring system, they were euthanised on 8 dpi. The four remaining animals (ID 31; 71; 73; 74) showed mild clinical signs of IAP first on 13–15 dpi which manifested as cyanoses of the ear tips and petechiae generalised over the body. Clinical signs exacerbated in three animals (ID 31; 73; 74) between 16 and 18 dpi with highly impaired general condition, high fever (up to 41.6 °C), anorexia, dyspnoea, skin pallor and ecchymosis on both ears. Due to fulfilment of the abortion criteria, two animals (ID 73; 74) were euthanised at 17 and 20 dpi, respectively. Antibiotic treatment with oxytetracycline and oral supplementation of glucose resulted in an improvement of clinical signs in the two remaining animals. However, recurrent occurrence of acute IAP was observed in both animals and they had to be euthanised on 41 (ID 31) and 62 dpi (ID 71), respectively. The course of the score points over time is displayed in Fig. 1.

### Haematological and blood chemistry findings

**Fig. 2:**
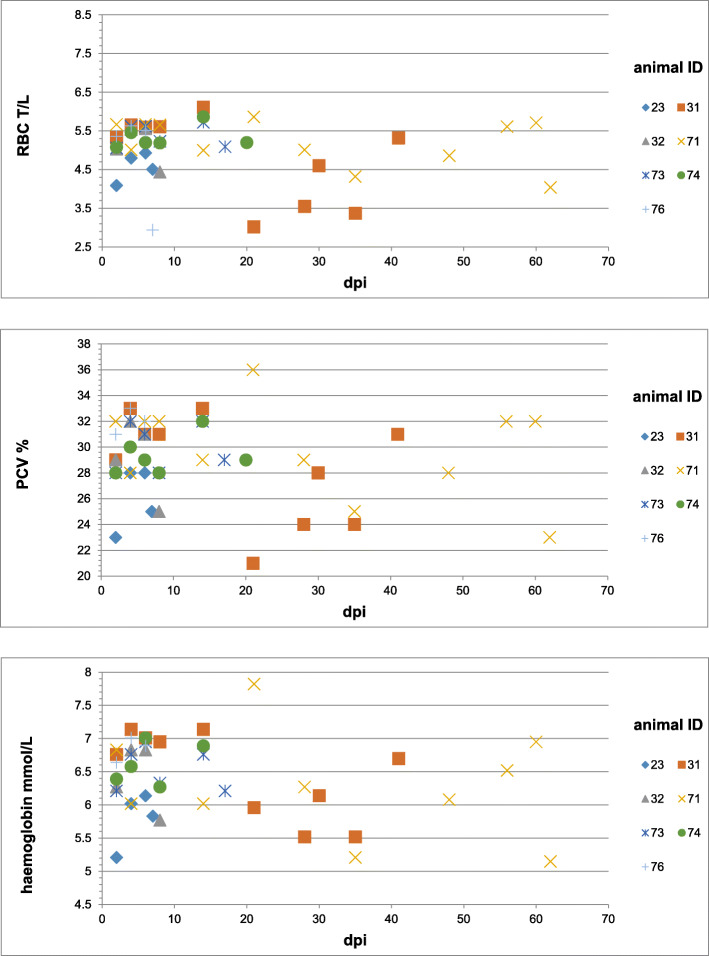
Anaemia outcome of experimental *M. suis* infection in splenectomised pigs. Red blood cell count (T/L), haemoglobin (mmol/L) and PCV (%) concentration of all seven animals

All animals developed a normochromic, normocytic anaemia at the time of acute clinical signs. RBC, haemoglobin and packed-cell-volume (PCV) concentrations from 0 dpi until euthanasia of the last animal (62 dpi) are shown in Fig. 2. Mean leukocyte counts increased from 2 to 6 dpi from 15.43 G/L (SD ± 4.35, SEM ± 1.65) to 22.27 G/L (SD ± 6.79, SEM ± 2.57). At the time of the first clinical signs at 7–8 dpi a decrease of the leukocyte count to an average of 16.98 G/L (SD ± 2.09, SEM ± 0.94) was detected. The highest leukocyte count was measured in animal ID 71 accompanied by severe clinical signs at 49 dpi with a value of 40.7 G/L.

With the onset of first clinical signs related to acute IAP, five (ID 23, 32, 73, 71, 76) out of the seven animals developed hypoglycaemia. Hypoglycaemia was also observed in the recurrent IAP attacks. The lowest glucose concentration of 0.2 mmol/L was recorded on 8 dpi (ID 76). At the time of acute IAP attacks increased bilirubin concentrations were recorded ranging from 3.25 μmol/L to 160.2 μmol/L. Urea concentrations increased in six out of seven animals (ID 23; 32; 71; 73; 74;76) at the onset of clinical signs. At the individual termination point of the experiment, serum iron concentrations decreased in all animals.

### M. suis blood and tissue loads

*M. suis* was first detected in the blood on 4 dpi in three animals (ID ID23, 74, 76) by qPCR. On day 6 all animals were *M. suis* positive. *M. suis* blood loads ranged between 2.2 × 10^3^ *M. suis*/mL on 4 dpi (ID23) and 9.6 × 10^9^ *M. suis*/mL on 8 dpi (ID 32).

As shown in Table 1, *M. suis* was found in all investigated tissue samples with the highest bacterial loads in the kidney (range: 1.76 × 10^10^ *M. suis*/g tissue (ID 32) - 5.98 × 10^11^ *M. suis*/g tissue (ID 31) and the lowest bacterial loads in the liver (range: 1.52 × 10^4^ *M. suis*/g tissues (ID 74) - 2.32 × 10^6^ *M. suis*/g (ID 73)).

**Table 1:** *M. suis* bacterial loads in the blood and in organs at the time of necropsy

ID	blood	lung	liver	kidney	brain	bone marrow	lymph nodes
	***M. suis***/ mL	***M. suis***/ g	***M. suis***/ g	***M. suis***/ g	***M. suis***/ g	***M. suis***/ g	***M. suis***/ g
**23**	3.60 × 10^9^	1.64 × 10^6^	2.14 × 10^6^	4.98 × 10^11^	9.06 × 10^5^	5.08 10^11^	6.26 × 10^11^
**31**	9.20 × 10^7^	3.48 × 10^6^	2.58 × 10^5^	5.98 × 10^11^	1.44 × 10^11^	8.54 × 10^4^	6.46 × 10^10^
**32**	9.58 × 10^9^	1.14 × 10^5^	5.80 × 10^5^	1.76 × 10^10^	4.20 × 10^7^	5.98 × 10^4^	7.94 × 10^9^
**71**	4.48 × 10^7^	4.64 × 10^6^	5.60 × 10^4^	2.06 × 10^10^	3.18 × 10^7^	2.08 × 10^6^	4.30 × 10^3^
**73**	2.38 × 10^9^	4.70 × 10^6^	2.32 × 10^6^	6.64 × 10^10^	3.12 × 10^5^	2.60 × 10^7^	3.54 × 10^3^
**74**	1.27 × 10^7^	2.40 × 10^5^	1.52 × 10^4^	2.18 × 10^11^	8.34 × 10^3^	2.40 × 10^3^	1.19 × 10^3^
**76**	8.68 × 10^9^	5.84 10 ^5^	1.22 × 10^6^	2.86 × 10^10^	3.58 × 10^11^	3.60 × 10^11^	2.54 × 10^11^

### Macroscopic findings

**Fig. 3:**
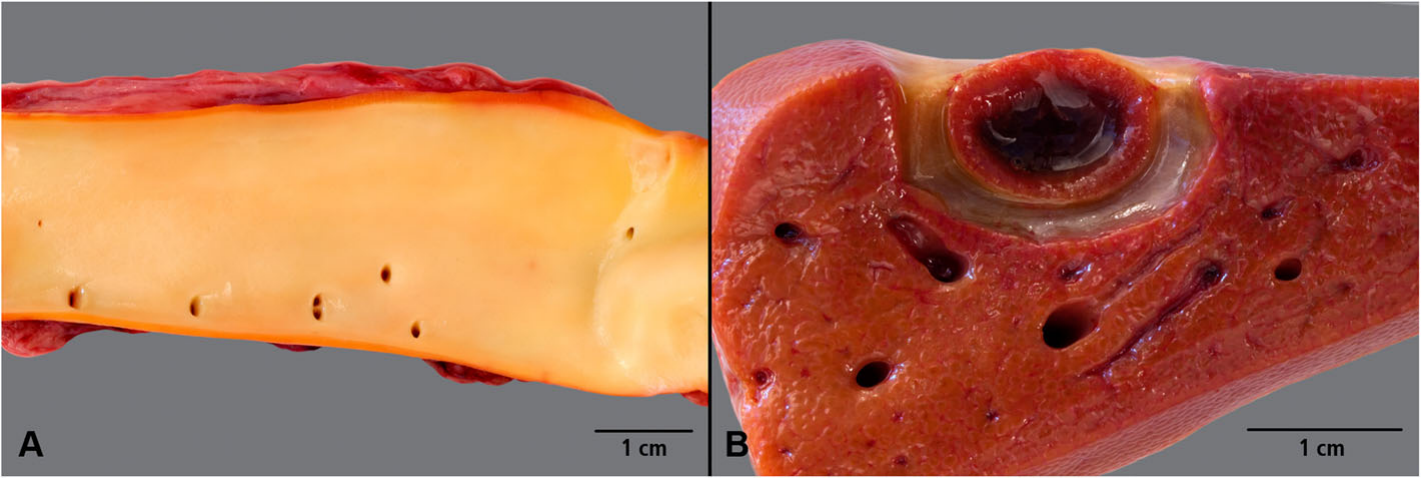
Macroscopic findings after experimental *M. suis* infection. Yellowish discoloration of the aorta (**A**), liver with oedema of the gall bladder bed (**B**)

Gross necropsy findings included severe icterus with yellowish discoloration of the skin and mucous membranes in two out of the seven animals (ID 73; 74). In addition, accumulation of watery translucent, icteric discoloured fluid was found in the abdominal cavity in animal 74. Signs of anaemia such as pale musculature were found in two animals (ID 71; 73). Both animals further presented severe swelling of the mediastinal and intestinal lymph nodes which were also reddened by blood resorption. Additionally, moderate to severe yellowish discoloration of the tunica intima of the aorta was observed in three animals (ID 32, 73, 74) (Fig. 3). Five animals showed macroscopic abnormalities of the liver. In addition to irregularly distributed herds with a white-yellowish colour and a diameter of up to 5 mm (32; 73; 74), one liver was very pale (ID 76). In two cases (ID 71; 74) a moderate to severe gallbladder oedema was also evident (Fig. 3).

Hyperaemia of the lung tissue and pulmonary oedema and emphysema were observed in three animals (ID 23, 32 and 76).

Macroscopically, changes of the kidneys were noticed in all animals. In addition to brightened hyperaemic tissue (71; 76), multiple and blurred whitish lesions with a diameter of up to 5 mm were observed, extending either over the entire renal tissue (21; 73; 74) or only over the renal cortex (31).

Macroscopic examination of other investigated organs (cerebrum, cerebellum, intestinal tract) revealed no obvious findings in all seven animals.

### Microscopic findings and detection of M. suis in Giemsa stained slides

**Fig. 4:**
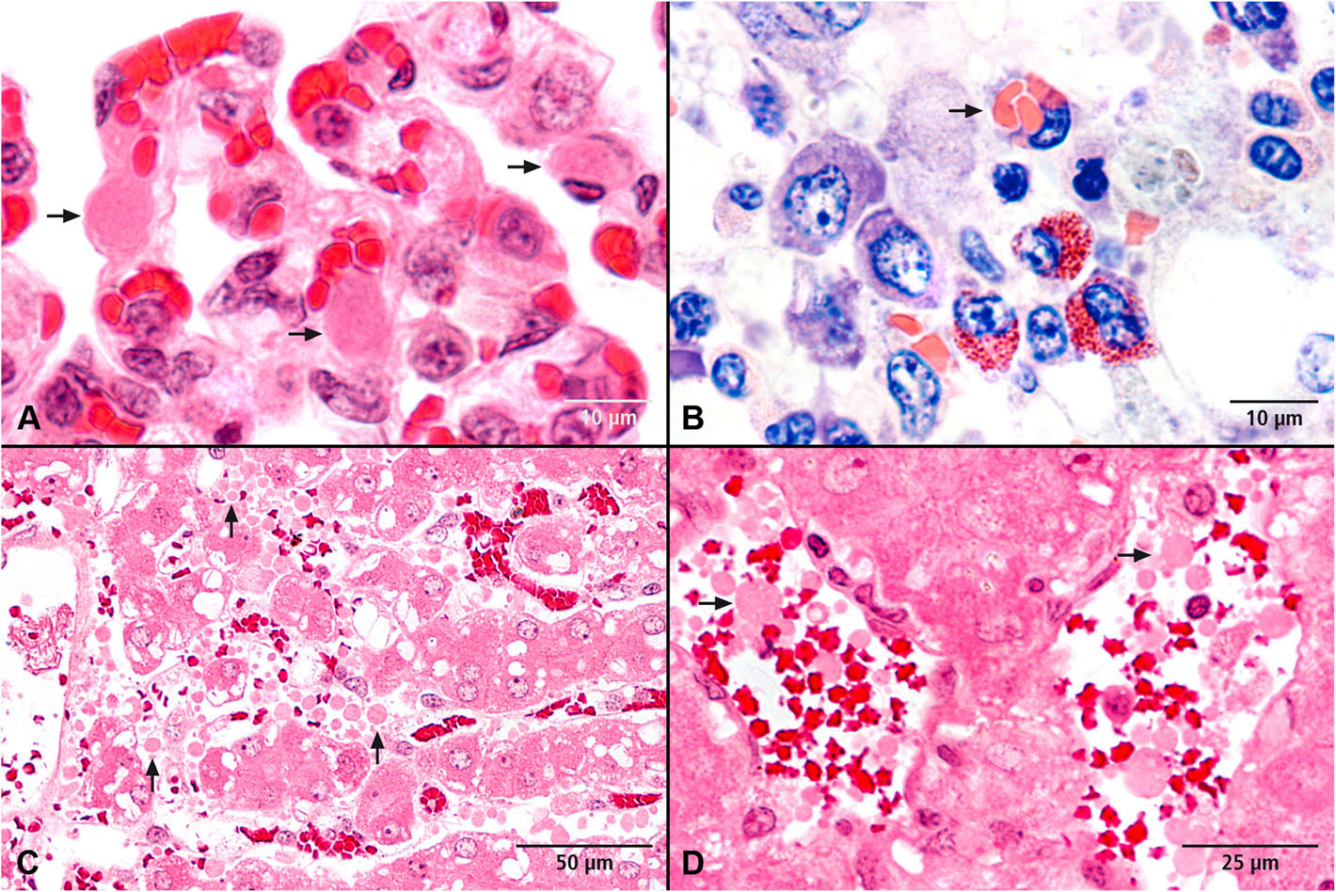
Histopathological changes after experimental *M. suis* infection. **A**) Lung with hyalin thrombi (arrows) in the alveolar capillaries; H&E stain. **B**) Bone marrow with erythrophagocytosis by a macrophage (arrow); Giemsa stain. **C**) Liver macrovascular steatosis, necrosis of centrilobular hepatocytes and atypical coagulation products (globular hyaline microthrombi) in the sinusoids (arrows) as an indicator of a disseminated intravascular coagulation; H&E stain. **D**) Liver with globular hyaline microthrombi in the sinusoids (arrows); H&E stain

Microscopic examination revealed low-grade nonspecific reactive interstitial hepatitis involving lymphocytes, plasma cells, eosinophilic and basophilic granulocytes as well as macrophages in three animals (ID 23; 71; 76). Additionally, in four animals (ID 73, 74, 31, 71) a multifocal erythrophagocytosis by Kupffer cells was detected and in two animals, haemosiderin was found in these macrophages (ID 23, 32). A total of three animals showed mild to severe oedema of the gallbladder bed (ID 23, 74, 71). In four animals (ID 71, 73, 74, 76) hyaline thrombi representing atypical coagulation products appeared in the hepatic sinusoids and vessels (Fig. 4). Centrilobular necrosis and periportal necrosis of the liver were found in ID 73 and ID 74, respectively. Additionally, steatosis of intact hepatocytes was found in both of those animals. Further, a high degree of dilatation of the portal lymph vessels was observed in some animals (ID 31, 32, 73, 74). In four of the seven animals (ID 31, 73, 74, 76), the moderate alveolar and interstitial pulmonary oedema and the dilatation of the lymphatic vessels was detected by histopathological examinations. Furthermore, the lungs of all seven animals revealed signs of severe blood clotting disorders. In detail, this was characterised by the presence of hyaline globules and thrombi in the alveolar capillaries (ID 71) (Fig. 4) and by the detection of fibrin in highly dilated alveolar capillaries (ID 76) and small pulmonary vessels (ID 31, 32).

Although macroscopic changes of the kidneys were found in all animals, only two kidneys showed histopathological alterations including nonspecific chronic inflammation of the connective tissue of the renal pelvis (ID 32) and non-purulent interstitial nephritis with dilatation of the lymph vessels (ID 31). Microscopic examination of the lymph nodes revealed signs of blood resorption (ID 71, 73), increased phagocytosis of the RBCs and the occurrence of siderophages (ID 31, 32, 71, 74, 76). The mesenteric lymph nodes of two animals showed follicular hyperplasia (ID 31) and sinus histiocytosis (ID 23), respectively. In two animals (ID 31, 74) fibrin was also found in the sinuses of different lymph nodes.

In addition, histopathological vascular changes could be detected in two animals. In animal ID 73 multifocal fibrin nets were found in medium-sized vessels of various organs. Focal endothelial cell loss with thrombus formation, mural fibrin insudation, mixed-cell infiltrates and acute bleeding could be observed in the *V. cava*. The animal ID 32 showed a pronounced circular fibrin exudation in the aorta with concomitant infiltration of macrophages and lymphocytes. A largely unchanged and haematopoietically active tissue with dominant erythropoiesis was found in sternal and femoral bone marrow (Fig. 4). Three animals (ID 23, 31, 71) showed erythrophagocytosis by macrophages in the marrow.

Microscopic examination of the brains revealed various findings. Multifocal eosinophilic nerve cell necrosis in the cerebral cortex (ID 23), multifocal low-grade acute bleeding and oedema in the leptomeninx (ID 76), multifocal high-grade perivascular plasma cellular and histiocytic infiltrates in the plexus of the fourth ventricle (ID 32) and endothelial cell swellings (ID 73) were found. Vasal and perivascular neutrophilic granulocytes and monocytes were detected in choroid plexus (ID 31, 73), leptomeninx (ID 71), cerebral cortex (ID 74) and subependymal in the lateral ventricle (ID 73).

**Fig. 5:**
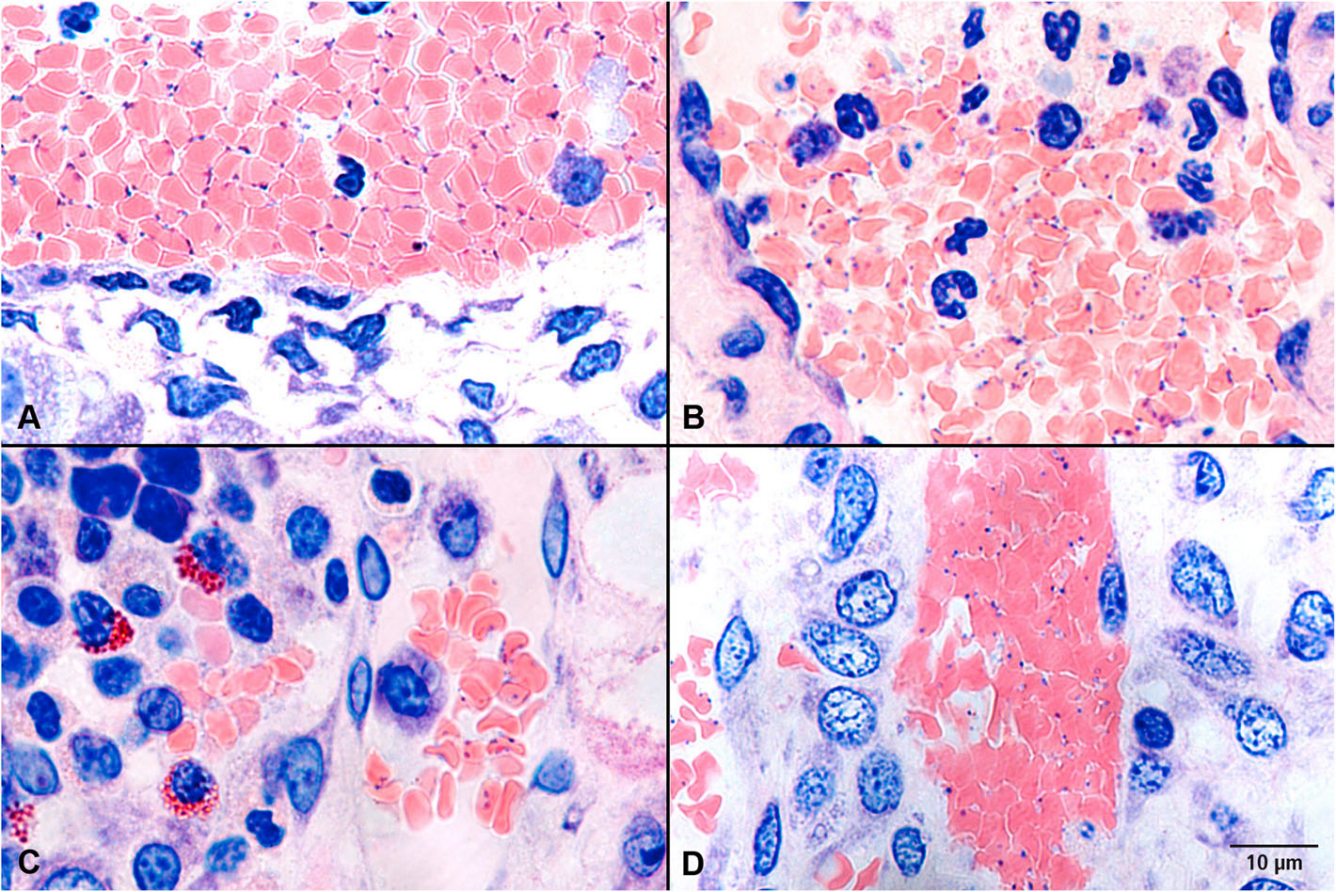
Detection of *M. suis* on RBCs in Giemsa stained slides. Images from liver (**A**), lung (**B**), bone marrow (**C**), and kidney (**D**)

In two out of the seven PCR positive animals *M. suis* was detected in Giemsa stained slides of all investigated organs. In the Giemsa staining *M. suis* was only associated with RBCs (Fig. 5)*.*

## Discussion

Due to the lack of in vitro cultivation system for *M. suis* challenge experiments are necessary to expand our knowledge on the pathogenesis of *M. suis* infections. Despite new insights into the pathobiology of *M. suis*, obtained from studies using modern molecular technology, including genomics and proteomics fairly little is known about *M. suis* target tissues and sequestration sites. Therefore, we conducted an experimental trail investigating lesions induced after *M. suis* infection and distribution of *M. suis* in different tissues.

Clinical signs of acute IAP were first observed on 7 dpi which contrasts with a shorter incubation period reported after inoculation with the highly virulent and RBC-invasive*M. suis* strain KI3806 [10]. The incubation period might vary due to virulence of the *M. suis* strains, infection dose or immune status of the animals [2]. Unlike Stadler et al. [10] where termination of the experiment had to be conducted during the first IAP attack on 6–8 dpi we were able to characterise clinical manifestation, haematologic alterations and *M. suis* blood loads in recurrent IAP attacks. Remarkably, no variation in the intensity of the clinical signs was observed between the individual attacks during the course of the experiment. Interestingly, the survival of the animals seemed to be associated with bacterial loads on 6 dpi as animals with a bacterial blood loads > 2.1 × 10^5^ *M. suis*/mL had to be euthanised already on 7 dpi or 8 dpi.

In accordance with previous studies a normochromic, normocytic anaemia as well as hypoglycaemia was found in all infected animals at the time of IAP attacks [10, 15, 17]. However, anaemia and hypoglycaemia were less severe compared to inoculation with the highly virulent *M. suis* strain KI3806 [10] what might be explained by differences in pathogenicity between *M. suis* strains or the bacterial loads which are negatively correlated with the aforementioned blood parameters [10]. Additionally, an increase of bilirubin concentration was observed at the time of acute IAP attacks. Next to haemolysis intra- or posthepatic disorders can result in increased bilirubin concentration. Increased urea concentration was recorded in the majority of animals at the time of IAP attacks. While a previous study [18] indicates that impairment of the kidney likely results from *M. suis* induced intravascular coagulation no glomerular thrombi were detected in the necropsied pigs. Another possible explanation for increased urea concentration might be fever occuring during acute IAP. The decrease of the iron concentration was likely a consequence of haemolysis as indicated by increased erythropoiesis in the bone marrow during microscopic examination and deposition of haemosiderin in macrophages of various organs. Alternatively, the iron decline might result from the metabolism of *M. suis* since proteome analysis revealed that ABC transporters specific for ferrichrome and haemin are expressed during acute IAP [19].

Skin alteration in terms of icterus and haemorrhagic diathesis (petechiae, urticaria) were evident in splenectomised pigs of the present study as early as 7 dpi. Although cutaneous manifestations resulting from a so far unknown pathogenesis have formerly thought to occur only in chronic stages of the disease, a more recent study described lesions from 17 dpi onwards in experimentally infected non-splenectomised pigs [10].

Despite numerous lesions no pathognomonic features for IAP were found in the present study which is in accordance with Dent et al. [20]. The most prominent macroscopic findings were in agreement with previous observations paleness and icterus of the skin, mucous membranes and organs [21–23]. A regular microscopic finding in various organs was disorder of blood coagulation in terms of thrombi in vessels and fibrin in the sinuses of different lymph nodes. Interestingly a previous study described disseminated intravascular coagulopathy (DIC) in the course of acute IAP [18]. Various mechanisms can be considered for the activation of intravascular coagulation processes. IgG autoantibody formation against the host’s RBCs is known to happen during acute IAP [24]. It is recognised that circulating antigen-antibody complexes can accumulate in the vascular walls of target organs such as the skin and result in an activation of the complement system, which can lead to platelet aggregation with formation of microthrombi and triggering of the coagulation cascade. Conformational transformations of the RBC phospholipid membranes can also cause changes in the coagulation cascade [25]. In the case of IAP this could occur due to increased eryptosis, which has also been described previously [25]. Another common finding was dilatation of the lymphatic vessels as an indicator of increased permeability of blood vessels leading to oedema in various organs (lung oedema, oedema of gallbladder bed, oedema in the leptomeninx). One possible explanation for permeability disorder might be a direct or indirect endothelial cell damage as previously described after *M. suis* infection [12]. As hyaline thrombi and other coagulation products were found in the vessels of lungs and liver the observed oedema might also result from coagulation disorders with subsequent circulatory disorders.

*M. suis* induced centrilobular necrosis of the liver was observed in one animal and has been described previously [18, 26]*.* In general, the most common cause of centrilobular necrosis is hypoxia of tissues. Hypoxia during IAP likely results either from anaemia or DIC [18]. The hyaline thrombi detected in hepatic sinusoids and vessels of four animals serve as indicator of DIC. Haemosiderin, a degradation product of haemoglobin was found in Kupffer cells in two animals which is a common finding of haemolytic anaemia and has been observed previously after *M. suis* infection [26, 27].

To summarise, the macroscopic and histologic lesions are likely to result from haemolysis with consecutive anaemia as well as disseminated intravascular coagulation and resulting organ impairment.

Antibiotic treatment did not result in clearance of the pathogen as indicated by permanent detection of *M. suis* in blood samples throughout the entire investigation period. Sequestration of haemotrophic mycoplasma in tissues has been proven in previous studies [28–30] and is discussed as the reason for chronicity of infection and failure of antibiotic treatment in clearing the infection. In a recent study an extended cell tropism of *M. suis* for endothelial cells was identified [12]. To investigate new potential host cells and sequestration of *M. suis* in tissues we sampled different organs and were able to detect *M. suis* in different quantities in the respective tissues. In contrast to *M. haemofelis* infected cats higher tissue loads were observed in the present study. In order to determine haemoplasma copy numbers in tissue other than expected from the blood supply Tasker et al. [28] have defined a ratio of expected haemoplasma quantity (resulting from the bacterial load at the time of sampling and the blood supply of the organs) and the actual haemoplasma concentration.

Such calculation cannot be extrapolated to pigs as data on organ perfusion for pigs are lacking. However, if we adopt the equation from cats [28] a higher ratio of tissue copy numbers than expected due to the blood supply can be calculated in the present study for kidney (7/7), lung (2/7), brain (2/7) and liver (1/7). Next to the missing porcine blood supply data, the results must be interpreted cautiously as no established protocol for comparing blood and tissue bacterial copy numbers have been published so far. As *M. suis* was exclusively associated with RBCs in Giemsa stained slides our study indicated no evidence of so far unknown potential host cells. However, further investigations with methods showing a higher sensitivity (i.e. immunohistochemistry, in-situ-hybridisation) are certainly needed.

## Conclusion

In the present study tissue sequestration of *M. suis* and resulting macroscopic and microscopic lesions were investigated in experimentally infected pigs. *M. suis* was detected in various organs by qPCR, however no evidences of RBC independent sequestration in different organs were found. Observed gross-necropsy and histopathological findings in *M. suis* infected pigs are most probably resulting from anaemia caused by haemolysis together with disseminated intravascular coagulation and consequential effects on other organs.

## Methods

### Animals and study design

Seven 28-days old, *M. suis* qPCR negative female piglets (ID 23, 31, 32, 71, 73, 74, 76) [31, 32] were included in the study. The experimental protocol as well as all any procedures were officially approved by the Government Office of Upper Bavaria, Munich, Germany (authorization reference number 55.2–1-54-2532-87-12). Details of the experimental procedures and monitoring of infection have been described previously [10]. Briefly, 1 week after placement at the Clinic for Swine, the piglets underwent splenectomy according to the protocol of Heinritzi [33]. Pigs were infected subcutaneously, (1.5 mL; 2.0 × 10^7^ *M. suis*/mL; *M. suis* field strain K323/13) 1 week after splenectomy. Clinical assessment was performed according to a score system described by Stadler et al. [10] with some modifications (Table 2). A score exceeding three points/day was determined as acute IAP attack which was treated with oxytetracycline (20 mg/kg body weight/q24h) and glucose (35 g/L drinking water). In the case of high fever (> 42 °C) animals received metamizole (30 mg/kg body weight). When reaching the stop criteria, (i.e. a score of > 3 remaining constant over 48 h despite antibiotic treatment, sustained fever of > 40 °C as well as impaired general health and anorexia), the affected animal was euthanised intravenously with pentobarbital (45 mg/kg body weight).

**Table 2:** Clinical score system

organ/ tissue	score Points	clinical signs
ears	0	no alterations
	1	mild cyanosis
	2	moderate cyanosis and necrosis
skin	0	no alterations
	1	moderate pallor
	2	generalised petechiae
	3	icterus
body temperature	0	< 40 °C
	1	40–42 °C
	2	> 42 °C
behavior	0	no alterations
	1	reduced
	2	apathy
feed intake	0	no alterations
	1	reduced
	2	anorectic
respiration	0	no alterations
	1	mild dyspnoe
	2	severe dyspnoe

EDTA-anticoagulated blood and serum samples were collected every 2 days for the first 8 dpi and afterwards once a week until the end of the trial planned on 90 dpi.

### Haematological and biochemical blood analysis

The following haematological parameters were recorded from EDTA-anticoagulated blood using the Scil Vet™ ABC tool (Scil Animal Care Company GmbH, Viernheim, Germany): RBC count (T/L), haemoglobin concentration (mmol/L), packed-cell-volume (PCV) (%), mean corpuscular haemoglobin (MCH), mean corpuscular volume (MCV), mean corpuscular haemoglobin concentration (MCHC) and leukocyte count (G/L). Glucose (mmol/L), iron (μmol/L), urea (mmol/L) and bilirubin (μmol/L) concentrations were determined by a Hitachi C 311 Chemistry Analyzer (Roche, Mannheim, Germany) from serum samples.

### Pathological and histopathological investigations

Necropsy and a macroscopic pathological examination were performed on all animals. Tissues of the following organs were collected during necropsy: aorta; bone marrow; brain (cerebrum and cerebellum); gall-bladder; heart; intestines; kidney; liver; lung; mesenterial lymph nodes; pancreas; skeletal muscle (*M. triceps*); skin; stomach; *V. cava*. After sampling, tissues used for histopathological examination were immediately transferred to 4% paraformaldehyde and fixed at 4 °C for 24 h. Tissues were embedded in plastic and stained according to Giemsa as well as with haematoxylin-eosin-phloxin (HEP).

### Detection and quantification of M. suis in blood and tissue samples

*M. suis* blood and tissue loads were determined by specific quantitative real-time PCR (qPCR) as described elsewhere [31, 32]. For blood samples, DNA extraction started with a preliminary treatment of 200 μl EDTA-anticoagulated blood using a modified lysis buffer (10 mM Tris pH 7.5, 5 mM MgCl_2_, 30 mM sucrose, 1% (v/v) Triton-X-100) followed by purification with the GenElute™ Bacterial Genomic DNA Kit (Sigma-Aldrich, Steinheim, Germany) according to the manufacturer’s instructions [32]. For tissue samples (bone-marrow; brain; liver; lung; lymph nodes; kidney) 200 mg of each specimen were mixed with 800 μl PBS and subsequently were homogenised with the FastPrep® FP 120 cell disruptor (Thermo Savant, Qbiogene Inc., Illkirch, France). One PBS control was carried with each ten samples to check for possible cross-contaminations.*M. suis* DNA was quantified afterwards by real-time PCR using the StepOne System™ (Applied Biosystems®) and primers specific for the *M. suis msg*1 [31].

### Statistics

Data were analysed with Excel (Microsoft® 2016) and the statistical software IBM SPSS, Statistics 22.0 (IBM Corporation, USA). Haematological and biochemical parameters were evaluated by means of descriptive statistics which included the calculation of the mean, standard deviation (SD) as well as standard errors of the mean (SEM). Differences in mean blood parameters between 0 dpi and the consecutive sampling days were investigated by a paired sample t-test.

## Data Availability

The datasets used and/or analysed during the current study are available from the corresponding author on reasonable request.

## Abbreviations

DIC: disseminated intravascular coagulopathy
dpi: days post infection
HEP: haematoxylin-eosin-phloxin
IAP: Infectious anaemia in pigs
*M. suis*: *Mycoplasma suis*
MCH: mean corpuscular haemoglobin
MCHC: mean corpuscular haemoglobin concentration
MCV: mean corpuscular volume
PCV: packed-cell-volume
qPCR: quantitative real-timepolymerase-chain reaction
RBC: red blood cell
